# Metabolic Profiling Reveals Sphingosine-1-Phosphate Kinase 2 and Lyase as Key Targets of (Phyto-) Estrogen Action in the Breast Cancer Cell Line MCF-7 and Not in MCF-12A

**DOI:** 10.1371/journal.pone.0047833

**Published:** 2012-10-24

**Authors:** Nadja Engel, Jan Lisec, Birgit Piechulla, Barbara Nebe

**Affiliations:** 1 University of Rostock, Department of Cell Biology, Rostock, Germany; 2 Max Planck Institute for Molecular Plant Physiology, Potsdam-Golm, Germany; 3 University of Rostock, Department of Biochemistry, Rostock, Germany; Massachusetts General Hospital, United States of America

## Abstract

To search for new targets of anticancer therapies using phytoestrogens we performed comparative metabolic profiling of the breast cancer cell line MCF-7 and the non-tumorigenic breast cell line MCF-12A. Application of gas chromatography-mass spectrometry (GC-MS) revealed significant differences in the metabolic levels after exposure with 17ß-estradiol, genistein or a composition of phytoestrogens within a native root flax extract. We observed the metabolites 3-(4-hydroxyphenyl)-lactic acid, cis-aconitic acid, 11-beta-hydroxy-progesterone, chenodeoxycholic acid and triacontanoic acid with elevated levels due to estrogen action. Particularly highlighted were metabolites of the sphingolipid metabolism. Sphingosine and its dihydro derivate as well as ethanolaminephosphate were significantly altered after exposure with 1 nM 17ß-estradiol in the cell line MCF-7, while MCF-12A was not affected. Treatment with genistein and the flax extract normalized the sphingosine concentrations to the basic levels found in MCF-12A cells. We could further demonstrate that the expression levels of the sphingosine metabolizing enzymes: sphingosine-1-phosphate kinase (Sphk) and lyase (S1P lyase) were significantly influenced by estrogens as well as phytoestrogens. The isoform Sphk2 was overexpressed in the tumorigenic cell line MCF-7, while S1P lyase was predominantly expressed in the non-tumorigenic cell line MCF-12A. Importantly, in MCF-7 the weak S1P lyase expression could be significantly increased after exposure with 10 µM genistein and 1 µg/ml root flax extract. Here, we present, for the first time, an analysis of metabolic response of phytoestrogens to breast cancer cell lines. The contrasting regulation of sphingolipid enzymes in MCF-7 and MCF-12A render them as preferred targets for future anticancer strategies.

## Introduction

Phytoestrogens are plant-derived phytochemicals which can react like the endogenous steroid hormone 17ß-estradiol because of their structural similarity. Especially flavonoids, such as daidzein and genistein, initially isolated from soybean, are well studied phytoestrogens with the potential to prevent cancer development and progression [1]. It was shown that some phytoestrogens e.g. genistein mediate estrogenic effects at low concentrations (<10 µM) whereas higher concentrations (≥10 µM) cause anti-estrogenic activity [2]. This biphasic role for genistein has been studied primarily in the human breast cancer cell line MCF-7 [3], [4]. Genistein at high concentrations has the ability to induce growth arrest and apoptosis in ER-positive cell line MCF-7 most likely by inhibiting the intrinsic tyrosine kinase activities of growth factor receptors [5]. However, the reason why endogenous estrogen hormones or synthetic xenoestrogens can increase breast cancer risk and phytoestrogens appear to exert a preventive effect is still not fully understood.

Until now, research was focused on genome-wide gene expression profile studies to enlighten the transcriptional regulation properties of phytoestrogens. Only recently, one group evaluated the transcriptional responsiveness of breast cancer cells to soy phytoestrogens using a whole-genome microarray based approach [6]. They identified 334 differentially expressed genes after treatment with 18.5 µM genistein or 78.5 µM daidzein which belong to completely different metabolic pathways. In addition to transcriptional analysis, downstream mechanisms, often referred to as non-genomic estrogenic pathways, became more and more in focus during the search for new phytoestrogen targets.

Here, we report for the first time on the influence of phytoestrogens on the metabolome of breast cancer cells. To this end, comparing GC-MS analyses of MCF-7, a well established breast cancer cell line, and MCF-12A, a non-tumorigenic epithelial breast cell line, allowed to distinguish between the metabolic features of breast cancer cells in contrast to their healthy counterparts. Both cell lines were positive for ERα and –ß expression [7]. To gain deeper insights in the mode of action of phytoestrogens and how they can diminish the proliferation-promoting action of 17ß-estradiol, we treated the cells with 17ß-estradiol, genistein and a natural blend of phytoestrogens extracted from the native root of *Linum usitatissimum* [8], [9]. This flax extract is composed of a variety of classical isoflavones and lignanes which potentially leads to synergistic effects on breast cancer cells in such a combination. This approach allows comparing tumor-relevant metabolic effects of cancerous cells and normal cells under tumor-promoting (17ß-estradiol) and tumor-regressing (phytoestrogens) modes and, therefore, enables the identification of new targets for anticancer treatment.

The combination of transcriptomics, metabolomics and intelligent pattern recognition methods for tumor metabolism recently allowed advances in breast cancer profiling [10], [11]. As a result, metabolic pathways, like the biosynthesis of bile acids, extracellular matrix synthesis or sphingolipid metabolism, are progressively gaining research attention. Bioactive sphingolipids play a key role in cancer progression, especially for proliferation, migration and tamoxifen resistance [12], [13]. The balance between ceramide, sphingosine and sphingosine-1-phosphate (and also their dihydro derivates) is thought to regulate cellular processes, including cell survival, growth and differentiation. Especially sphingosine-1-phosphate (S1P) which is controlled by S1P kinases (isoforms: Sphk1, Sphk2; biosynthesis) and S1P lyases (degradation) is a well established pro-survival molecule [14]. It was shown that S1P promotes the migration of MCF-7 cells, suggesting a role for Sphks in metastasis [15]. The biological effects of S1P are mediated by five specific G-protein-coupled receptors located on the cell surface (named S1P_1,2,3,4,5_). The functional S1P_1_ antagonist FTY720 and inhibitor of Sphk1 activity decreases breast cancer proliferation and metastasis in mouse models [12]. Several growth factors and steroid hormones, such as TGF-ß and 17ß-estradiol could be related to up-regulation of Sphk1. The role of Sphk2 in cancer remains somewhat unclear and the irreversible conversion of S1P to ethanolamine and hexadecanol by S1P lyase is unstudied so far.

## Materials and Methods

### Cell Culture and Treatment Conditions

Cell culture conditions of cell lines MCF-7 and MCF-12A, purchased from ATCC (www.atcc.org/) were described previously [7]. At a sub-confluence of approximately 80% the cell monolayer was washed with PBS and adapted to phenol-red-free Dulbecco’s Modified Eagle’s Medium (PAA Laboratories GmbH, Germany) with 10% charcoal stripped fetal bovine serum (PAN Biotech GmbH, Germany) for 48 hours to avoid unspecific stimulation of endogenous hormones in the serum. Treatments with the flax extract (L; final concentrations: 0.01, 1 and 50 µg/ml), the phytoestrogen genistein (G; 5,7,40 trihydroxyisoflavone; final concentration: 1, 10 and 100 µM) and 17ß-estradiol (E; final concentration: 1 nM), last two purchased from Sigma, Germany, was carried out for 48 hours. Extract preparation from flax roots of *Linum usitatissimum* (L) was described previously [8], [9]. Lignan/isoflavone contents of the flax extract according to Luyengi extract preparation were about 1.25–4.25 mg/g fresh weight (0.125%–0.425%) [8]. As negative control substances the respective vehicle (C; final concentration: 0.1%) was used in the same manner.

### Flow Cytometric Measurements of Cell Proliferation and Apoptosis

Flow cytometric measurements and calculation of proliferation and apoptosis was done as described in detail [7], [16].

### Metabolic Profiling via GC-MS

The metabolite profiles were measured by gas chromatography–mass spectrometry (GC–MS). For each sample, 200,000 MCF-7 and 460,000 MCF-12A cells were harvested with 0.05% trypsin-0.02% EDTA, washed three times with ice-cold PBS and cell pellet was frozen in liquid nitrogen after centrifugation (14,000 rpm, 4°C, 2 min). Sample extraction and derivatization followed the procedure described previously [17]. Metabolite signals were obtained from raw data and compared against a reference database using the TargetSearch package [18]. Some samples were removed after inspection of their chromatograms due to overall lower peak intensities, leaving four to six replicates per group (all samples of the same genotype and subjected to the same treatment). Out of all putative metabolic traces only 106 were retained in the resulting raw data matrix that met the following criteria: (i) the peak was present in all samples of at least one replicate group (genotype/treatment combination); (ii) the median peak height exceeded a value of 250 (well above the detection limit); (iii) the peak mass spectra showed a similarity of >75% to the respective library entry and a retention time deviation of <2s (can be considered as correctly identified). All data were log10-transformed to improve normality and normalized to show similar median peak intensity within replicate groups as described previously [19].

### Western Blot

After treatment with phytoestrogens for at least 48 h the cells were trypsinized, washed with PBS and lysed in ice-cold lysis buffer (Bio-Plex Cell Lysis Kit, Bio-Rad, USA). Cells were homogenized by brief sonification at 4°C and centrifuged at 10,000 g for 2 min at 4°C. Protein concentrations of the supernatants were estimated by Bradford protein assay [20] and verified by Coomassie staining [21] so that equal amounts (10–30 µg) of total soluble protein could be separated by SDS-PAGE and blotted on PVDF membranes, subsequently. After the protein transfer membranes were blocked with 5% skim milk in Tris-buffered saline (TBS) and washed six times in TBS. For protein detection primary antibodies (Sphk1: sc-48825; Sphk2: sc-22704; S1P-lyases: sc-67368, all from Santa Cruz, USA) were incubated overnight at 4°C followed by a labeling with a horseradish peroxidase (HPR)-conjugated secondary antibody (Dako, Glostrup, Denmark) for 1 hour at room temperature. Protein signals were visualized by using SuperSignal West Femto Chemiluminescent Substrate (Pierce Biotechnology, Rockford, USA) for detection of peroxidase activity from HRP-conjugated antibodies (Thermo Fisher Scientific Inc., Rockford, USA). Band intensity was analyzed densitometrically with the Molecular Imager ChemiDoc XRS and Image Lab 3.0.1 software (Bio-Rad, USA). Protein detection was repeated at least three times with individual prepared cell lysates from independent passaged cells.

### Immunofluorescence Staining

For immunofluorescence staining 1×10^5^ cells were seeded on glass cover slips (20×20 mm) and let them adhere for 24 hours. Thereafter treatment with phytoestrogens for 48 h and control substances followed as described above. Cover slips were washed twice with Dulbecco’s PBS without Ca and Mg, fixed with 4% paraformaldehyde for 15 min, washed again and permeabilized with 0.1% Triton X-100 for 15 min. After a washing step with phosphate buffered saline (PBS) cells were incubated with the primary antibody (see Western blotting procedure) in a dilution of 1∶50 at 4°C overnight. Afterwards cells were washed three times with PBS and incubated with 488Alexa Fluor-labeled secondary antibody (Molecular Probes, USA, 1∶100) for 1 hour at room temperature in the dark. After washing cells were incubated with DAPI (Roche Diagnostics GmbH, Germany) to stain nuclei for 15 min. Finally the cells were washed four times with PBS, embedded and stored in the dark at 4°C. Visualization and imaging was carried out with the Axio Scope.A1 fluorescence microscope (Carl Zeiss, Germany) using AxioVision Imaging Software 4.8.2.0 (Carl Zeiss, Germany). Notably, photomicrographs stained with the same primary antibody were taken at identical exposure times to guarantee comparable results.

### Live Cell Monitoring of Adhesion, Acidification and Respiration

Online monitoring of the adhesion, acidification, respiration under the influence of sphingosine-1-phosphate (S1P) and D-sphingosine (D-S) was performed with the Bionas® 2500 analyzing system with the metabolic chip SC 1000 (Bionas GmbH, Rostock, Germany) and the measurement software Bionas15002 CS1.47 [22]. Prior experiments, chips were cleaned with 70% ethanol for 10 minutes, washed with PBS and were adapted to the measurement medium for 5 minutes. Measurement medium was composed of DMEM without NaHCO_3_ (Invitrogen, Germany), 0.1% charcoal stripped fetal bovine serum (PAN Biotech GmbH, Germany) and 1% gentamycin (Ratiopharm, Germany), pH value 7.4 and sterile filtered. On each chip 2×10^6^ cells were seeded and let them adhere over night at 37°C and in 5% CO_2_ so that 80% sub-confluence on the sensor chips was reached. Bionas measurements were carried out with a pump rate of 56 µl/min for 24 hours [22], [23]. Within the first 4 hours cells could adapt to the new measurement medium. Thereafter cells were treated with the vehicle substance (control), 1 µM S1P (Sigma, Germany) or with 1 µM D-S (Sigma, Germany) for 20 h. Every measurement was repeated three times. Data set was evaluated and normalized with the software Bionas1500^2^ Data analyzerV1.07.

### Statistical Analysis

Flow cytometry, western blotting and immunofluorescence experiments were replicated at least three times with individual passaged cells and data sets were expressed as means ± standard deviations (SD). Statistical significance was determined by the student’s t-test (** P<0.001, * P<0.01).

All statistical tests on metabolite profiles have been conducted in R (www.r-project.org) using the respective functions. To facilitate comparison of treatment effects in MCF-7 and MCF-12A, two further normalization strategies have been followed for metabolic values, applying the following formula:
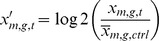


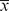
 the observed, normalized and mean value and m, g and t are metabolite, genotype and treatment, respectively. Basically this transformation expresses all values relative to a control group mean and centers the deviations on zero (due to log2). For 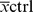 we choose either the control group mean of the same genotype as the sample (strategy one) or consistently the control group mean of MCF-12A (strategy two). While the first strategy removes any differences between the control samples of MCF-12A and MCF-7, therefore highlighting the treatment effects, the second strategy maintains between line differences. To investigate which metabolite levels are stronger affected in MCF-7 compared to MCF-12A after treatment with E, we conducted t-tests between control (C) and estradiol (E) treatment for both lines. Instead of selecting all metabolites showing non-significant P-values in MCF-12A and significant P-values in MCF-7, we decided to select candidates based on the P-value ratio of both tests being larger than 100. That means we also selected metabolites that had a (significant) P-value of 0.01 in MCF-12A if the observed P-value for MCF-7 was lower than 0.0001. Because this is rather intuitive than statistically sound, we confirmed this ranking by manual inspection of the data using boxplots. For principal component analysis we used the pcaMethods package [24], applying the nipals algorithm on pareto normalized data. For analysis of variance (ANOVA) we considered genotype and treatment as factors and allowing for interactions. The resulting P-values were Bonferroni corrected. Heatmaps were produced using the pheatmap package.

## Results and Discussion

### Determination of the Effective Phytoestrogen Concentrations

**Figure 1:**
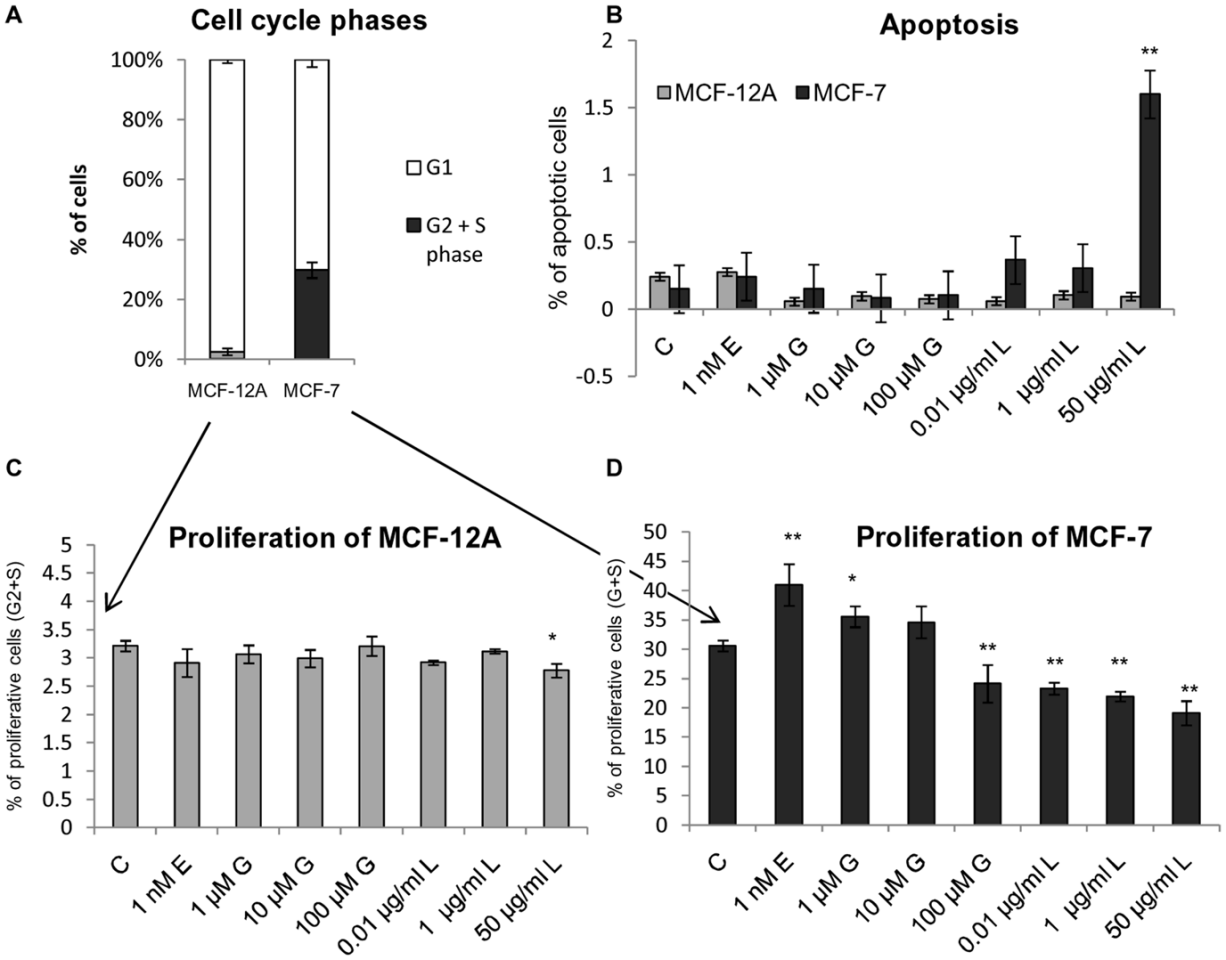
Cell cycle analysis to determine proliferation and apoptosis. Determination of effective 17ß-estradiol (E), genistein (G) and flax extract (L) concentration on the cancerous cell line MCF-7 and the non-tumorigenic cell line MCF-12A via cell cycle analysis by flow cytometry. Control (C) treatment was carried out with the vehicle ethanol (EtOH). **A**: Percentage distribution of cell cycle phases in both cell lines under un-treated conditions. Proliferative phases were summed as G2+S. Notably, the MCF-7 cells line harbors a more than 10fold greater proliferative potential with up to 30% cells in G2+S phase. **B–D**: Dose dependent effects of genistein and flax extract on apoptosis and proliferation (G2+S) on the cell lines MCF-7 and MCF-12A. All results are indicated in overall percentage. Mean ± SD values (n = 5−7). * = *p*<0.01; ** = *p*<0.001 as compared to EtOH control (unpaired *t* test).

Prior to metabolic profiling experiments we determined the most effective concentrations of genistein (G) and a native root flax extract (L) for our treatment conditions via cell cycle analysis in flow cytometry studies. First, the proliferative phases (S+G2/M) and the apoptotic rates (sub-G1) were calculated for the cell lines MCF-7 and MCF-12A under control conditions (Fig. 1A). The non-tumorigenic cell line MCF-12A showed low proliferative potential indicating that only 2–4% of all measured cells were in G2/M or S phase. In contrast, the tumorigenic cell line MCF-7 is marked by higher proliferative phases in the sum of about 27%. The apoptotic cells of both cell lines were below 0.5%, typical for a subconfluent, continuously growing cell culture (Fig. 1B). For validating the most effective concentrations of the phytoestrogens both cell lines were treated with either EtOH or 17ß-estradiol (C, E), both are regarded as control experiments, or three concentrations of genistein (G) or the flax extract (L) for 48 hours. Determination of the apoptotic rates revealed no significant alterations in both cell lines except at the highest concentration of the flax extract (50 µg/ml) for the MCF-7 (Fig. 1B). The detailed analysis (Fig. 1C) illustrated that the applied concentrations of genistein and flax extract are not cytotoxic, even for the non-tumorigenic cell line MCF-12A. This important to note because the MCF-12A cells are considered as a control cell line representing healthy, normal breast epithelial tissue. The proliferative rates in MCF-12A cells revealed that the highest concentration of the flax extract (50 µg/ml) lead to a moderate but significant impairment of the dividing activity of MCF-12A, while the other concentrations did not significantly affect the cell cycle phases of MCF-12A (Fig. 1C). Therefore, the highest concentration of the flax extract was excluded from further experiments because healthy tissue could be affected detrimentally. Treatment with E and showed stronger proliferative response in the cell line MCF-7 (Fig. 1D). The addition of 17ß-estradiol resulted in a strong increase of the proliferative phases of the estrogen-sensitive cell line MCF-7 (40–45%) indicating that nearly half of all MCF-7 cells were in the G2 or S phase. The application of G caused a concentration-dependent biphasic effect on the proliferation of MCF-7 cells, as already described [25]–[27]. The lowest concentration of 1 µM genistein yielded in 10–15% elevated rates of G2+ S phase compared to the control. The intermediate concentration of 10 µM showed no alteration of the cell cycle phases and the highest concentration of 100 µM led to a 20–30% reduction of the proliferative phases in MCF-7 *in vitro* (Fig. 1D). In contrast to the effects obtained after genistein application, the native flax extract significantly decreased proliferation of MCF-7 at all tested concentrations. The highest concentration of 50 µg/ml native flax extract resulted in the strongest inhibition (10–15%) of MCF-7 proliferation.

On the basis of these proliferation results we decided to use the low and intermediate concentrations of 1 µM and 10 µM of genistein for the following metabolic profiling experiments. As expected the low concentration revealed more estrogenic effect on MCF-7 cells. A concentration of 10 µM genistein could be reached in the human body after a phytoestrogen-rich diet and therefore support the transition to an anti-estrogenic action [28]. Since concentrations of 100 µM most likely do not appear in the human body these test concentrations are considered unphysiologically. Regarding the native flax root concentrations, 0.01 and 1 µg/ml were used for metabolic measurements because the highest concentration of 50 µg/ml caused a significant decrease in cell proliferation of MCF-12A. Apparently, the mixture of various phytoestrogens in a plant extract appears to be more effective for the inhibition of cell proliferation of MCF-7 than a moderately dosed, single isolated phytoestrogen like genistein.

### Metabolic Profiling of Mammary Epithelial Cell Lines (MCF-12A, MCF-7)

In a preliminary experiment, 200,000 MCF-7 and MCF-12A cells under normal culture conditions were harvested in five replicates. Comparison of the GC-chromatograms and mass spectra revealed drastic changes in the pool sizes of most metabolites, allowing a discrimination of tumorigenic and non-tumorigenic cells (data not shown). We further observed that metabolite levels were generally twice as high in MCF-7 compared to MCF-12A. This finding suggested that the MCF-7 cells are bigger or, less likely, contain proportionally larger amounts of primary metabolites, which are predominantly measured using GC-MS. We then determined the fresh weight of both cell lines and found MCF-7 cells to be approximately 2.3-fold heavier than MCF-12A cells (data not shown). Therefore, we decided to adjust the imbalanced metabolite levels by using 200,000 MCF-7 and 460,000 MCF-12A cells for every further metabolic measurement to ensure comparable results.

**Figure 2:**
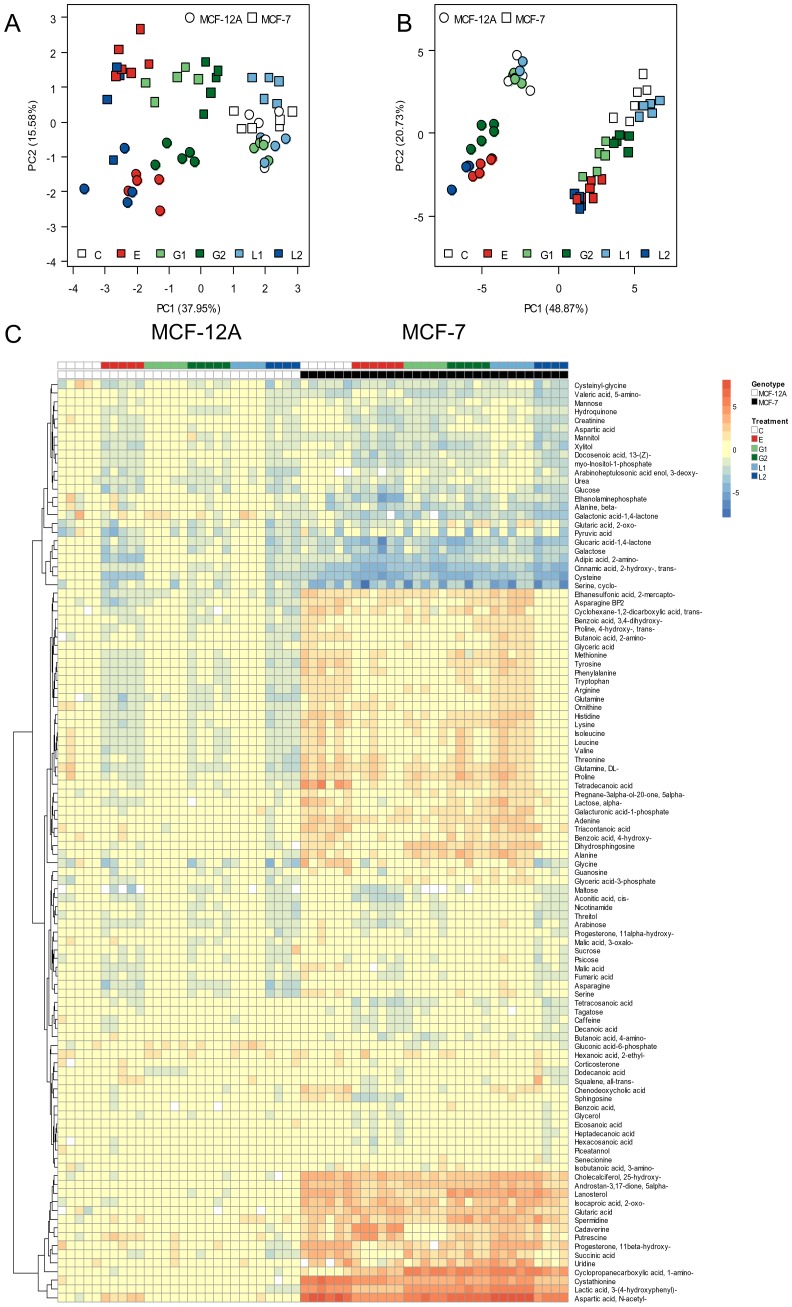
Heat map of metabolites comparing between MCF-12A and MCF-7. Principal component analysis (PCA) and heat map presentation of metabolite profiles from MCF-7 cells (squares) and MCF-12A (circles) treated with 0.1% EtOH (C, white), 1 nM 17β-estradiol (E, red), 1 µM genistein (G1, light green), 10 µM genistein (G2, dark green), 0.01 µg/ml *Linum usitatissimum* (L1, light blue) and 1 µg/ml *Linum usitatissimum* (L2, dark blue) for 48 hours. **A**: For log10-transformed values the main sources of variance are cell type (PC1, 49%) and treatment (PC2, 21%). **B**: For data expressed relative to the respective control samples the main sources of variance are treatment (PC1, 38%) and treatment within cell type (PC2, 16%). **C**: Heat map presentation of all identified metabolites by GC-MS based profiling under the treatment with 17ß-estradiol (E), genistein (G) and flax extract (L) in the cell lines MCF-12A and MCF-7 (see color bar for scale; blue color indicates down regulated metabolites; red color indicates up regulated metabolites). The data were normalized to the untreated control of MCF-12A to identify significantly altered metabolites in the tumorigenic cell line MCF-7. Metabolites were grouped according to cell line and each treatment condition (indicated with specific colors in the upper panel) and hierarchically clustered. Correlation coefficients were calculated by applying the Pearson algorithm using R software.

Subsequently to sample preparation, we could detect 106 metabolites that were identified based on retention times, characteristic ions and mass spectra, comprising mainly amino acids, organic acids and mono- and disaccharides. Initially, we applied principal component analysis (PCA) on the data to evaluate the major differences between the individual groups. As shown in Fig. 2A most of the variance is attributed to differences between the two cell lines (PC1, 49%), while treatment effects also contribute strongly (PC2, 21%). The differences between the lines are not caused by differences in cell weight. In contrast to our preliminary experiment we observed about 40% of all metabolites to show higher levels in MCF-12A compared to MCF-7. This was expected as we controlled for the total sample amount by cell number (see above). It can be seen that higher concentrations (E, L2, G2) generally modulate the metabolic profile of both lines stronger, while cells treated with lower concentrations (L1, G1) tend to closer resemble the metabolic profile of the control samples. An interesting exception is the treatment with G1, which shows no effect in MCF-12A but does show an effect on MCF-7. To render treatment effects more comparable between cell lines, we expressed metabolic profiles relative to the control samples of the respective cell line (cf. Material and Methods). This procedure removed most genotypic differences and allowed to checking if metabolic changes of similar treated samples were consistent or different between MCF-7 and MCF-12A. As can be seen in Fig. 2B, metabolic changes due to different treatments were not contrasting between the two cell lines but do show differences. Differences in metabolic profiles were further quantified by an analysis of variance (ANOVA) for each metabolite including the two factors genotype and treatment as well as an interaction term (Fig. S1). These results confirm the PCA by showing that most metabolites are found to be significantly different between genotypes (74 metabolites with Bonferroni corrected P-values <0.05) and treatments (82 metabolites).

To give a more intuitive representation of differences for individual metabolites a heatmap of metabolic levels was established (Fig. 2C). To optimally visualize genotypic and treatment effects in one plot, all metabolite intensities were normalized to the respective mean values of the MCF-12A controls (0.1% EtOH) since these data represent the metabolite levels of the unaffected, normal epithelial breast tissue cell line. A log2-transformation centers the values around zero, such that values of one and two indicate a two and four-fold increase compared to the control. Minus one and two indicate a two and four-fold decrease, respectively, compared to the control. Up- and down-regulated metabolites in comparison to MCF-12A are indicated by red and blue colors, respectively. As expected several metabolite levels were up-regulated in the breast cancer cell line MCF-7 under both, treated and untreated conditions in contrast to MCF-12A, with N-acetyl-aspartic acid, 3-(4-hydroxyphenyl)-lactic acid and cystathionine (dark red color) being the most prominent examples. We found metabolites significantly down-regulated in MCF-7 cells like cyclo-serine, 2-hydroxy-trans-cinnamic acid and cysteine (blue color).

While per se differences between the metabolism of cancerous and non-cancerous cells were investigated previously [29], [30] we focused our work on the metabolic alterations in these cell lines treated with 17ß-estradiol and phytoestrogens. Consequently, we searched for metabolites which were significantly influenced in the tumor line MCF-7 and not in the non-tumorigenic cell line MCF-12A after an exposure of 1 nM 17ß-estradiol. We ranked all metabolites according to their stronger alteration in MCF-7 versus MCF-12A after exposure of 1 nM 17ß-estadiol (Table 1). Sphingosine was ranked first and followed by the 3-(4-hydroxyphenyl)-lactic acid, 1-amino-cyclopropanecarboxylic acid, succinic acid, chenodeoxycholic acid and N-acetyl-aspartic acid. The ranking of the 29 metabolites fulfilling our criteria is given in Table 1 and box plots of their normalized levels can be found in Fig. S2. The 29 compounds are annotated to different cellular pathways, e.g. lipid metabolism, amino acid metabolism, steroid hormone synthesis, indicating that different targets were addressed by 17ß-estradiol treatment in cancer cells. Astonishingly, strongly effected metabolites appeared in lipid and steroid biosynthetic pathways. Furthermore, we noticed that three metabolites of the sphingolipid pathway (sphingosine, ethanolaminephosphate, dihydrosphingosine) were part of the ranking and, therefore, potentially main targets of 17ß-estradiol in the breast cancer cell line MCF-7 (highlighted in Table 1).

**Table 1:** List of the ranked metabolites.

No.	Metabolite	Related pathway	KEGG/pubchem no.
**1**	**Sphingosine**	**Lipid metabolism**	**C00319/cid: 5280335**
2	3-(4-hydroxyphenyl)-Lactic acid	Glycolysis	C01432/cid: 612
3	1-amino-Cyclopropanecarboxylic acid	Amino acid metabolism	C01234/cid:535
4	Succinic acid	Tricarboxyclic acid cycle (TCA)	C00042/cid: 1110
5	Chenodeoxycholic acid	Lipid metabolism	C02528/cid: 5645
6	N-acetyl-Aspartic acid	Amino acid metabolism	C01042/cid:97508
7	Cystathionine	Amino acid metabolism	C00542/cid: 834
**8**	**Dihydrosphingosine**	**Lipid metabolism**	**C00836/cid: 91486**
9	11-beta-hydroxy-Progesterone	Steroid hormone synthesis	C05498/cid: 92750
10	2-amino-Butanoic acid	Amino acid metabolism	Cid:6657
11	All-trans-Squalene	Steroid hormone synthesis	C00751/cid: 638072
12	Cis-Aconitic acid	Tricarboxyclic acid cycle (TCA)	C00417/cid: 643757
13	Threitol	Sugar alcohols	C16884/cid:169019
14	Corticosterone	Steroid hormone synthesis	C02140/cid: 5753
15	5-alpha-Androstan-3,12-dione	Steroid hormone synthesis	C00674/cid:3943
16	Triacontanoic acid	Lipid metabolism	Cid: 10471
17	Cadaverine	Amino acid metabolism	C01672/cid: 273
18	Malic acid	Tricarboxyclic acid cycle (TCA)	D04843/cid:525
19	Creatinine	Amino acid metabolism	C00791/cid: 588
20	Lanosterol	Steroid hormone synthesis	C01724/cid:4861
21	Piceatannol	Stilbenoid, diarylheptanoid and gingerol biosynthesis	C05901/cid:667739
22	4-hydroxy-, trans-Proline	Amino acid metabolism	C01015/cid:5810
23	Glutaric acid	Fatty acid metabolism; lysine degradation	C00489/cid:743
**24**	**Ethanolaminephosphate**	**Lipid metabolism**	**C00346/cid: 1015**
25	Caffeine	Purine metabolism; calcium signaling pathway	D00528/cid:2519
26	11-alpha-hydroxy-Progesterone	Steroid hormone synthesis	C03747/cid:6508
27	Galactonic acid-1,4-lactone	Ascorbate and aldarate metabolism	C01040/cid:97165
28	4-hydroxy-Benzoic acid	Amino acid metabolism	C00156/cid: 105001
29	5-amino-Valeric acid	Amino acid metabolism	C00431/cid:3720

Ranking of the 29 metabolites affected stronger by 17ß-estradiol in the tumorigenic cell line MCF-7 compared to the non-tumorigenic cell line MCF-12A. Related pathways and the KEGG and/or PubChem number are given.

**Figure 3:**
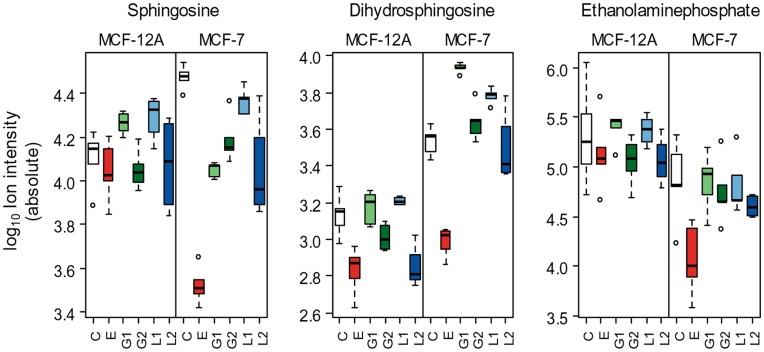
Alterations in sphingolipid metabolites. Boxplots of absolute metabolite levels for sphingosine, dihydrosphingosine and ethanolaminephosphate under the treatment of 17ß-estradiol (E), genistein (G) and flax extract (L) in the cell lines MCF-12A and MCF-7. Each box plot shows the median, and upper and lower quartiles. Outlier values are indicated by individual data points.

In Fig. 3 these three metabolites were analyzed in detail regarding all treatment conditions. Sphingosine was found in higher concentrations in the cell line MCF-7 under control conditions but after exposure 17ß-estradiol the levels were significantly reduced. The treatment with genistein as well as with the phytoestrogen mixture in form of the flax extract normalized the sphingosine levels in MCF-7 while the amounts in MCF-12A were not significantly affected. Similar results were obtained for dihydrosphingosine and ethanolaminephosphate with the exception that genistein and the flax extract could not normalize the expression of dihydrosphingosine in the cell line MCF-7 to gain the level of MCF-12A under control conditions, like it is was observed for sphingosine and ethanolaminephosphate. Our experiments clearly demonstrate that metabolites of the sphingolipid metabolism are one of the main targets for the action of 17ß-estradiol and phytoestrogens with similar structural properties like genistein.

### Expression of Sphingosine Kinase and Lyase is Influenced by Phytoestrogen Action

**Figure 4:**
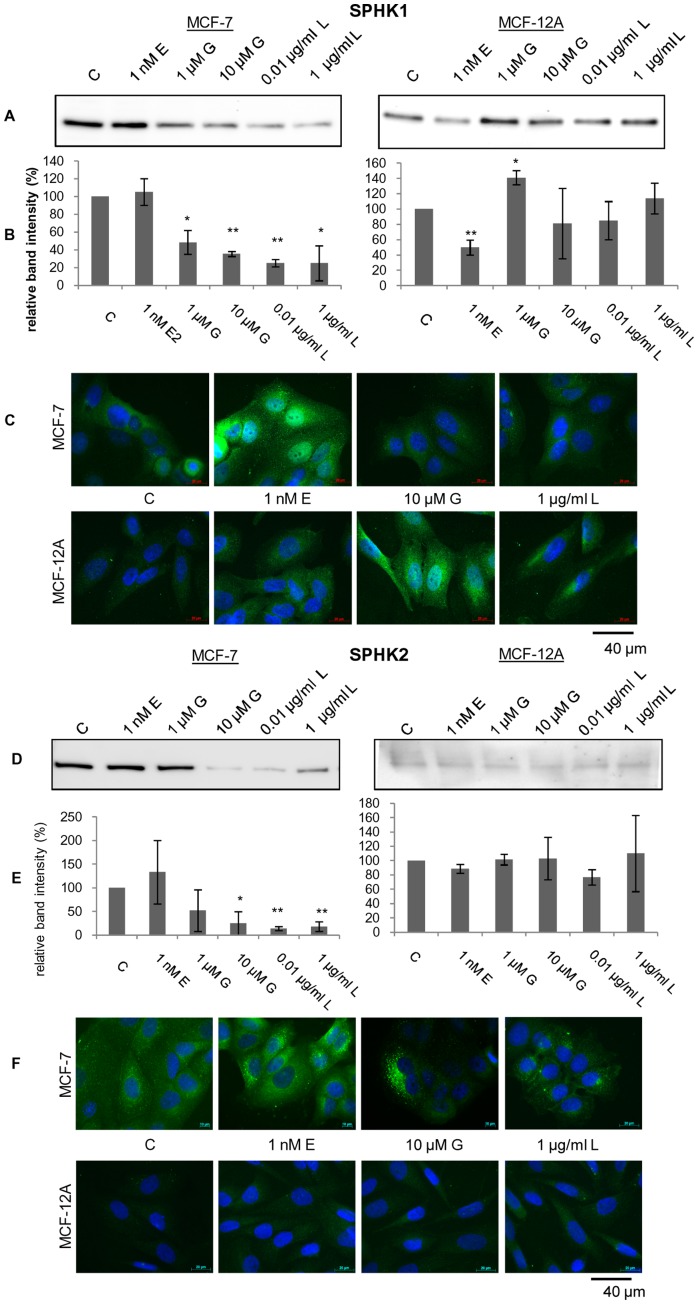
Expression regulation of Sphk1 and Sphk2. Western blotting (**A**, **D**), quantification of western blotting results (**B**, **E**) and immunofluorescence staining (**C**, **F**) of the sphingosine-1-phosphate kinase isoform 1 and 2 (Sphk1; Sphk2) expression level after 48 h exposure to 17ß-estradiol (E), genistein (G) and the root flax extract (L) at different concentration in the cell lines MCF-7 and MCF-12A. Western blotting and immunofluorescence staining were carried out with same primary antibody and were repeated at least three times with individual passaged cells. Single representative western blot and fluorescence images were displayed. SPHK expression in the immunofluorescence pictures was taken with a constant exposure time of 5.6 s for Sphk1 and 2.0 s Sphk2 (green); nucleus (blue). Mean ± SD values (n = 3−5). * = *p*<0.01; ** = *p*<0.001 as compared to EtOH control (unpaired *t* test).

**Figure 5:**
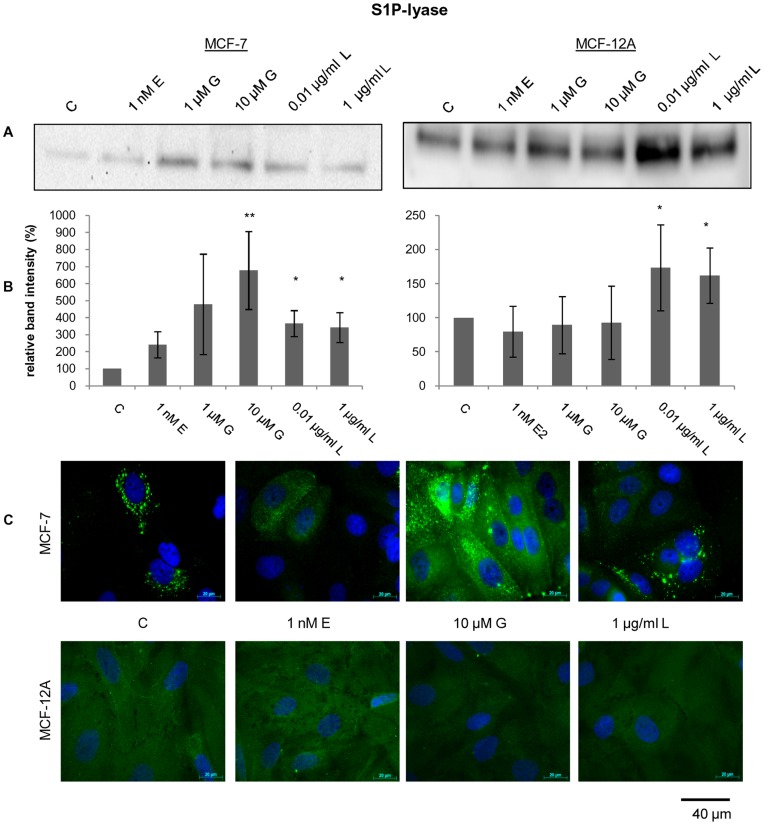
Expression regulation of Sphingosine lyase. Western blotting (**A**), quantification of western blotting results (**B**) and immunofluorescence staining (**C**) of the sphingosine-1-phosphate lyase (S1P lyase) expression level after 48 h exposure to 17ß-estradiol (E), genistein (G) and the root flax extract (L) at different concentration in the cell lines MCF-7 and MCF-12A. Western blotting and immunofluorescence staining were carried out with same primary antibody and were repeated at least three times with individual passaged cells. Single representative western blot and fluorescence images were displayed. S1P lyase expression in the immunofluorescence pictures was taken with a constant exposure time of 2.3 s. S1P lyase (green); nucleus (blue).Mean ± SD values (n = 3−5). * = *p*<0.01; ** = *p*<0.001 as compared to EtOH control (unpaired *t* test).

The metabolites sphingosine, dihydrosphingosine and ethanolaminephosphate are linked with each other via enzymatic steps including several enzymes. An overview of the sphingolipid metabolism is indicated in a modified KEGG pathway and schematic representation (Fig. S3, S4). Alterations in the protein content and distribution of three involved enzymes (Sphk1, Sphk2, S1P lyase) were analyzed by western blotting and immunofluorescence staining. Western blotting experiments revealed a significant decrease in the amount of Sphk1 and Sphk2 enzyme in the breast cancer cell line MCF-7 after 48 h exposure with G and L in a concentration dependent manner (Fig. 4A, B; Fig. 5A, B). Especially treatment with the flax extract resulted in a 2–3fold reduction of both kinases. Furthermore, we could observe a slight increase of Sphk1 and Sphk2 expression in MCF-7 after treatment with 1 nM 17ß-estradiol. In MCF-12A Sphk1 expression was decreased nearly by half after treatment with 17ß-estradiol and phytoestrogen exposure revealed boosted Sphk1 amounts (Fig. 4B). In contrast, western blots of MCF-12A cells showed poor, nearly undetectable expression rates of Sphk2 even when 20–30 µg soluble protein was loaded on the SDS-gel (Fig. 4D). Negligible alterations in the expression of Sphk2 were measured in MCF-12A after treatment with 17ß-estradiol and phytoestrogens (Fig. 4E). All western blot results were confirmed by immunofluorescence stainings with identical antibodies (Fig. 4C; Fig. 4F). Proteins of interest were secondary labeled with AlexaFluor488 (green color). Additionally, nuclei were stained with DAPI (blue) for better local orientation of the protein expressions within the cell. To guarantee quantitative comparable results we have ensured that photographs were taken with identical settings, e.g. exposure time. Representative pictures of Sphk1 expression in MCF-7 and MCF-12A were displayed in Fig. 4C. As in the western blots already indicated, phytoestrogens are able to decrease Sphk1 expression in the breast cancer cell line MCF-7 and enhance its expression in the non-tumorigenic cell line MCF-12A (Fig. 4C). When strong and constitutive expression was reached the cell nuclei were overlaid with the green dye indicating current protein biosynthesis at the surface of the rough endoplasmic reticulum. We conclude that 17ß-estradiol and phytoestrogens have opposite effects on the Sphk1 expression in MCF-7 and vice versa for MCF-12A. For Sphk2 we obtained similar results in the cell line MCF-7 (Fig. 4D). In contrast Sphk2 is expressed weakly in MCF-12A like it was shown by western blot result previously (Fig. 4E). Nearly no green fluorescence is visible inside the cell only the blue-glowing nuclei can be mentioned. These findings led us to the assumption that SPHK1 is expressed in cancerous as well as non-tumorigenic cells while SPHK2 is overexpressed in MCF-7 and, therefore, an important regulator of sphingosine phosphorylation.

The degradation of sphingosine-1-phosphate (S1P) to phosphoethanolamine and (2E)-hexadecenal is mediated by S1P lyase. Western blot analysis revealed a weaker expression in MCF-7 than in MCF-12A (Fig. 5A). But after exposure with genistein and the flax extract the amounts of S1P lyase increased dramatically in MCF-7. Up to 4–5 fold higher levels were reached, especially after treatment with 10 µM genistein. Similar results were obtained in immunofluorescence staining experiments. The strongest fluorescence in MCF-7 cells was seen after exposure with 10 µM genistein which correlated with significant elevated enzyme levels in western blot (Fig. 5B). In the non-tumorigenic cell line MCF-12A we observed constant expression levels of S1P lyase after treatment with 17ß-estradiol and genistein while both concentrations of the flax extract increased the S1P lyase amount up to 30% (Fig. 5B). While most results were confirmed by immunofluorescence staining, no confirmation with the western blotting results was observed in experiments with 10 µM genistein and 1 µg/ml flax extract (Fig. 5B, C). These experiments suggests that Sphk2 and S1P lyase may be useful targets for cancer therapy drugs, as decreasing or increasing its expression during tumorigenesis may help to regulate cell proliferation.

### Online Monitoring of Cellular Metabolism during Stimulation with Sphingolipids

**Figure 6:**
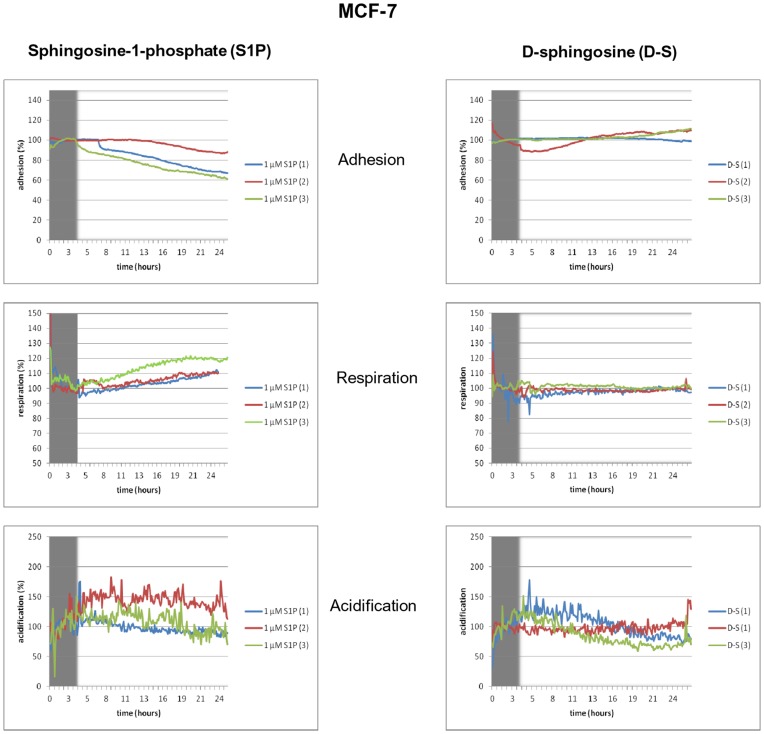
Online monitoring of cell metabolism. Online monitoring of cell metabolism in the cancerous cell line MCF-7 after exposure with 1 µM sphingosine-1-phosphate and 1 µM D-sphingosine. Displayed were the percentage of the standardized and normalized rates of cell adhesion (impedance), respiration (O_2_ consumption) and extracellular acidification in a period of 24 h. Displayed were three individual replicates. Grey shadowed area marks the adaption phase (4 h) of the cells to the new conditions.

To prove the influence of S1P and sphingosine on cell metabolism we used the Bionas® 2500 analyzing system and Bionas® metabolic chip SC1000 to monitor existing adhesion/impedance, O_2_ consumption (respiration) and the extracellular acidification of living MCF-7 cells under the exposure of either 1 µM S1P or D-sphingosine (D-S) in the course of 24 hours (Fig. 6). The exposure with S1P reduced the adhesion in all three replicates up to 40%. The respiration was increased in all cases whereas the acidification rates are subject to large fluctuations and in two measurements was slightly increased. As expected the treatment with D-S revealed completely different results for these three parameters. Sphingosine itself is known for its apoptotic and anti-proliferative affects [13]. Treatment with D-S revealed a significant increase of cell impedance/adhesion in two cases, while the respiration rates were not influenced. The acidification rates were lowered in two cases. These online monitoring studies regarding three relevant parameters of cell metabolism are reflecting the influence of the sphingolipids on the estrogen-receptor positive breast cancer cell line MCF-7. Despite the fact that flow cytometry measurements of proliferation and apoptosis indicated for no great alteration after exposure with neither S1P nor D-sphingosine the more sensitive method with the Bionas system confirmed the contrary effects of both sphingolipids on MCF-7 cells. Exposure with S1P caused greater acidification rates in the surrounding medium, while D-sphingosine lowered these levels. Therefore, we concluded that energy production by glycolysis was reduced and less lactate was produced. Apparently higher acidification rates in cancer cells are primarily traced to the lactate production and secretion, primarily. It is known that higher fluxes through glycolysis and increased lactate production are associated with tumor progression even when oxygen levels are abundant [29]. In addition the boosted respiration rates after treatment with S1P were indicating higher energy production via oxidative phosphorylation in the TCA (citric acid cycle) cycle. Taken together S1P exposure caused higher glycolysis as well as TCA fluxes in MCF-7 and therefore increasing the energy production dramatically. Lowered impedance of MCF-7 cells after exposure with S1P does not consequently imply that cells lose their contacts to the surface but changing shape and a reduced formation of focal adhesion might explain that effect as well. Conversely, the opposite reactions are applying for the rising impedance after treatment with D-sphingosine.

### Conclusions

Our metabolic profiling reveals members of the sphingolipid pathway as one of the main targets of estrogen and phytoestrogen action. A summary of the relationship between the three highlighted sphingolipids and their metabolic functions are shown in Fig. S4. The hormone 17ß-estradiol and the phytoestrogens execute contrasting reactions on the Sphks. While 17ß-estradiol increased the expression of Sphks in the cancerous cell lines MCF-7, the expression in non-tumorigenic cell line MCF-12A was lowered. The phytoestrogens react in the opposite way: higher expression in MCF-12A und significant decrease in MCF-7. This finding let us draw the conclusion that the exposure with phytoestrogens in higher concentration (genistein >10 µM; root flax extract >1 µg/ml) decreases tumor progression signaling via sphingolipids while normal tissue was not or positively stimulated. Furthermore, the degrading pathway of S1P to phosphoethanolamine by the reaction of the S1P lyase was primarily promoted by the addition of phytoestrogens in tumorigenic cell line MCF-7. The enhanced conversion to phosphoethanolamine could let to evaluated antitumor activity so that phytoestrogens have the ability to influence both, the enzymatic anabolic as well as the degrading step of S1P [31]. In addition, the expression of S1P lyase in the non-tumorigenic cell line MCF-12A was significantly up regulated under control conditions. It seems likely that normal mammary epithelial cells prevent extracellular S1P signaling by degrading this lipid before it reaches the receptors at the cell surface or the intracellular space, respectively [32]. Consequently, S1P lyase emerged as a possible target for anti-cancer research and production of effective chemotherapeutic agents.

## Supporting Information

Figure S1 **Histograms of Bonferroni corrected P-values obtained in a two way ANOVA with the factors genotype and treatment for absolute values of all 106 metabolic traits.** The upper panel shows the number of P-values significant at α = 0.05 for each factor and the interaction term, respectively, while the lower panel indicates the strength of the observed effects by presenting the data in log-scale. To this end, a comparable number of metabolic levels is significantly altered due to genotype or treatment, with genotypic effects showing generally lower P-values. However, this is partly due to the different number of levels for the factors (genotype: 2, treatment: 6).
(TIF)

Figure S2 **Overview of all mentioned metabolite boxplots.** Boxplots of all metabolites mentioned the ranking (Table 1) under the treatment of 17ß-estradiol (E), genistein (G) and flax extract (L) in the cell lines MCF-12A and MCF-7. Plot layout and colors are similar to Figure 3.
(TIF)

Figure S3 **Sphingolipid pathway in detail.** KEGG pathway of the sphingolipid metabolism in *Homo sapiens* (Entry no.: map00600) overlaid with boxplots of the detected metabolites.
(TIF)

Figure S4 **Overview of cellular sphingosine-1-phosphate regulation.** Scheme highlighting the relationship between regulation mechanisms of sphingosine metabolism in MCF-7 vs. MCF-12A.
(TIF)
