# Supplementary figures and images for: Metabolic Profiling Reveals Sphingosine-1-Phosphate Kinase 2 and Lyase as Key Targets of (Phyto-) Estrogen Action in the Breast Cancer Cell Line MCF-7 and Not in MCF-12A
Source: PLoS One. 2012 Oct 24;7(10):e47833. doi: 10.1371/journal.pone.0047833 (PMC3480432; doi:10.1371/journal.pone.0047833)

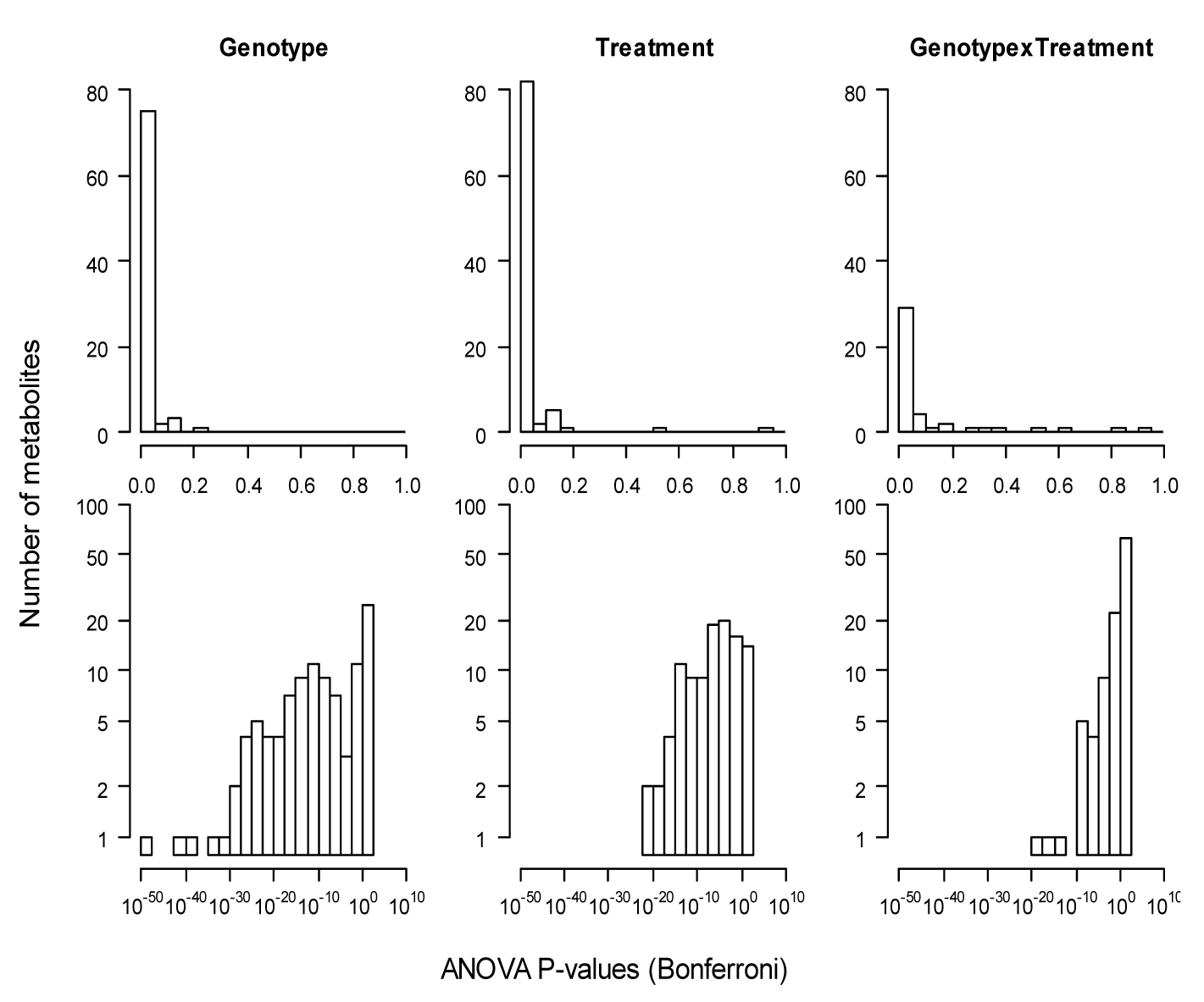

Supplement: Figure S1 — Histograms of Bonferroni corrected P-values obtained in a two way ANOVA with the factors genotype and treatment for absolute values of all 106 metabolic traits. The upper panel shows the number of P-values significant at α = 0.05 for each factor and the interaction term, respectively, while the lower panel indicates the strength of the observed effects by presenting the data in log-scale. To this end, a comparable number of metabolic levels is significantly altered due to genotype or treatment, with genotypic effects showing generally lower P-values. However, this is partly due to the different number of levels for the factors (genotype: 2, treatment: 6). (TIF) [file pone.0047833.s001.tif]

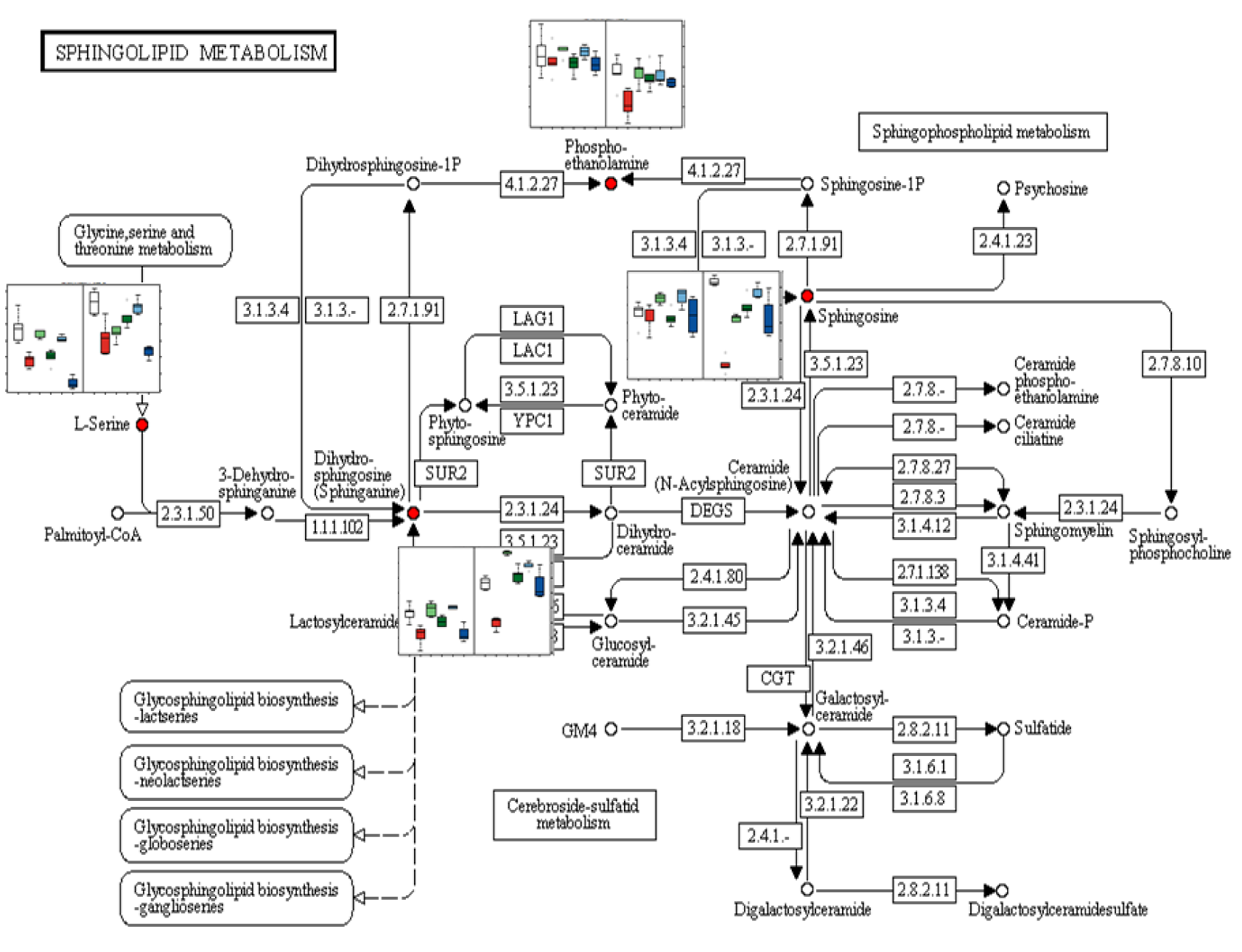

Supplement: Figure S3 — Sphingolipid pathway in detail. KEGG pathway of the sphingolipid metabolism in Homo sapiens (Entry no.: map00600) overlaid with boxplots of the detected metabolites. (TIF) [file pone.0047833.s003.tif]

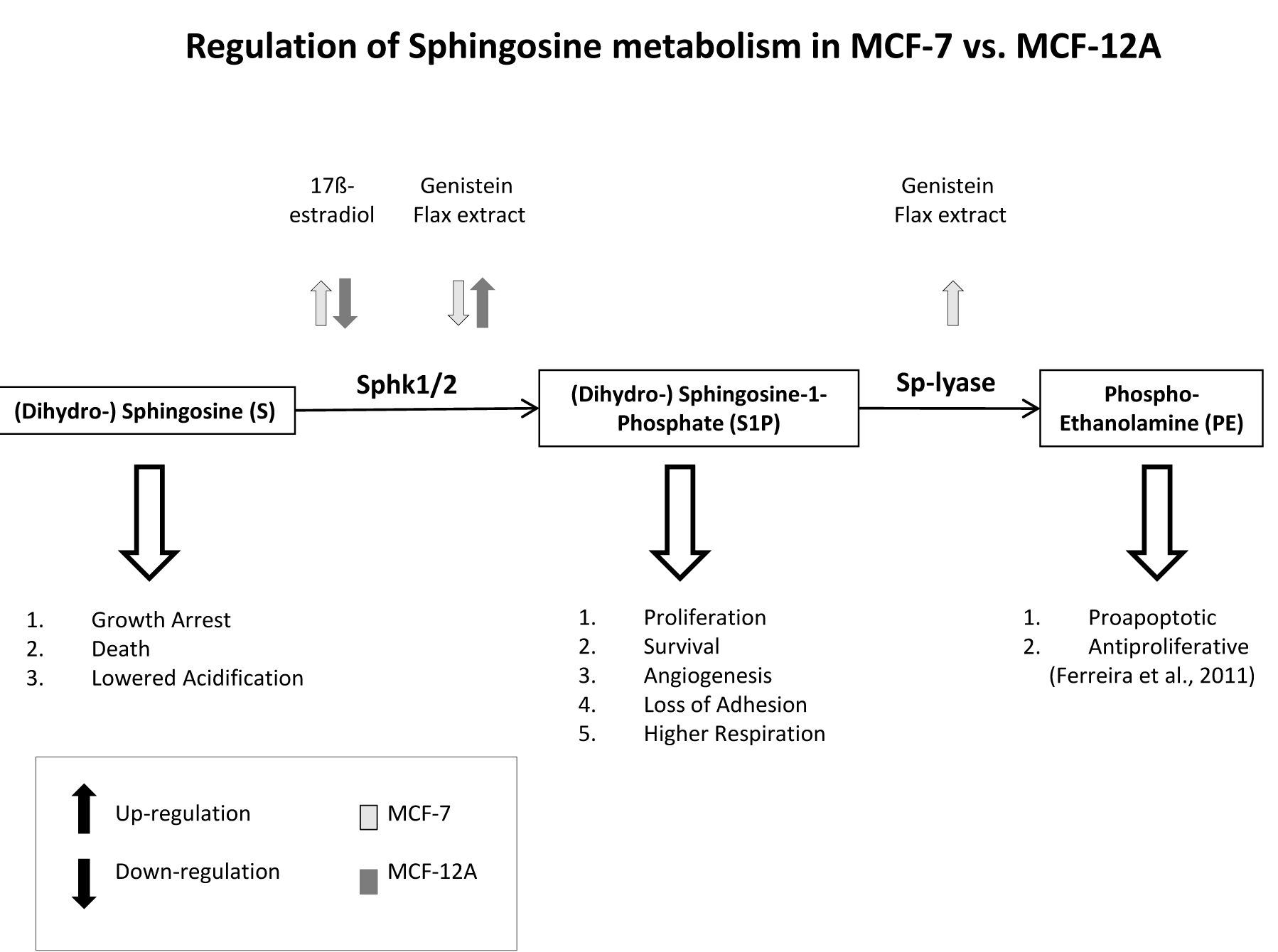

Supplement: Figure S4 — Overview of cellular sphingosine-1-phosphate regulation. Scheme highlighting the relationship between regulation mechanisms of sphingosine metabolism in MCF-7 vs. MCF-12A. (TIF) [file pone.0047833.s004.tif]
